# Socioeconomic Characteristics, Purchasing Preferences and Willingness to Consume Organic Food: A Cross-Location Comparison of Nine Cities in Central Ecuador

**DOI:** 10.3390/foods11243979

**Published:** 2022-12-08

**Authors:** Carlos Moreno-Miranda, Christian Franco-Crespo, Isabel Pachucho, Karla Uño, Ana Gordillo, Jacqueline Ortiz

**Affiliations:** 1 Faculty of Food Engineering and Biotechnology, Technical University of Ambato, Av. Los Chasquis and Río Payamino, 180150 Ambato, Ecuador; 2 Agricultural Economics and Rural Policy Group, Wageningen University and Research, Hollandseweg 1, 6706 KN Wageningen, The Netherlands; 3 Department of Consumer Research, Ecuadorian Fruit and Vegetable Processing Industry Inphec, Ambato Industrial Park-CEPIA, 180150 Ambato, Ecuador

**Keywords:** consumer behavior, healthier diet, household income, logit model, survey research

## Abstract

Agriculture worldwide faces the need to reduce chemical pesticides and produce healthier food. In Latin America, research on the organic food sector primarily focuses on supply. Consumption analysis is crucial for providing information about customers’ needs. This paper aims to analyze the Ecuadorian organic food sector, which is an interesting case for investigating the relationship between willingness to consume organic food and socioeconomic factors. To this end, 382 consumers were surveyed. The study applied a logit regression analysis to assess the role of socioeconomic factors in the willingness to consume organic food. Radar diagrams depict the percentage of respondents who checked a particular reason for choosing or refusing organic food. A cross-location comparison analysis was applied to identify differences between locations within the Central Ecuador region. Results reveal that Ecuadorian consumers’ awareness rate of organic food is only 53.5%. Regarding organic food’s price, 24% of consumers perceive it to be overly expensive. Based on strengthening the supervision of organic food production, various channels should be used to promote organic food consumption and facilitate the recognition of available organic food.

## 1. Introduction

Agriculture worldwide must reduce chemical pesticides use to provide healthier food. Overexploitation and ecosystem pollution have been linked to conventional agriculture. Excessive use of fertilizers and pesticides pollutes the environment and harms human health [1]. Organic food production has emerged as a viable alternative to polluting food production methods [2]. Organic agriculture is performed in 179 nations on 43 million hectares or 0.98 percent of the world’s agricultural area [3]. Organic farming is a viable industry in the United States and Canada, with sales increasing by roughly 4% yearly [4]. With an annual average growth rate of 12%, Europe leads the world in organic food consumption [5]. Kulak et al. [6] pointed out that consumption analysis of organic food is crucial for leading actors and stakeholders by providing information about customers’ needs and helping in decision-making and formulating strategies. However, research on the organic food demand and consumption in Latin American countries still needs to be made available.

Ecuador is gaining more engagement in organic food production. There are approximately 41,838 hectares of organic farming [7]. Furthermore, Ecuadorian state policies stimulate rural development through organic and environmentally friendly agriculture [8]. Several programs focusing on organic and agroecological farming incorporate women to support gender equality because it makes women’s contributions more visible [9]. On the demand side, several studies, e.g., Carrión Bósquez et al. [10] or Diaz-Basantes et al. [11] point out that 60% of Ecuadorian consumers consider purchasing organic foods for their health [12]. Organic products are becoming more popular as people are more aware of environmental issues [13], and new marketing channels, such as short market circuits, have approached organic food to consumers [14]. However, Tulla et al. [15] report that relatively few consumers are well-informed about the benefits of consuming organic products, and determinants influencing consumer preferences for organic food still need to be discovered.

This study focuses on the Ecuadorian organic food industry, which provides an interesting case study examining the connection between socioeconomic and informational factors and organic food adoption. First, organic food’s perishable nature limits its distribution channels [16]. Second, players push organic food consumption through a variety of mechanisms [17]. Third, the government still needs to stabilize organic food prices, and the fragmentation of local markets continues to hamper the sector’s growth [18]. The study utilized a framework to examine the effect of socioeconomic and informational factors on willingness to consume organic food.

The study contributes to the analytical and empirical challenges of organic food willingness to consume. It also substantially contributes by merging socioeconomic and informational elements in a logit model. So, according to Boas et al. [19] and Moreno-Miranda et al. [20], socioeconomic measures in food consumption are essential for evaluation, and Halbe and Adamowski [21] assert that concurrent treatment of variables in willingness to consume assessments poses drawbacks. Thus, trade-offs between the determinants constitute a significant problem in assessment approaches [22,23]. The lack of findings in existing research undermines the credibility of willingness to consume assessments [24]. Therefore, the final contribution of this research is the cross-location comparison analysis of the willingness to consume organic food between locations within Central Ecuador.

The paper is structured as follows. Section 2 provides the theoretical framework and hypothesis. Section 3 outlines the research design and methodology. Section 4 describes the data. Section 5 focuses on the research findings and presents the discussion. In the final section, we discuss the research implications and conclusions.

## 2. Literature Review

Many empirical studies on healthy food consumption have examined the factors determining consumers’ organic meal choices. It depends on the methodological framework; such investigations can be categorized into two kinds. Using a discrete choice model, the first set of studies addresses factors influencing consumers’ organic food choices. The second set of studies employed multiple methodologies to explore the underlying mechanisms controlling organic food preference.

Kuhar and Juvancic [25] specified an ordered probit framework to analyze the leading causes explaining the repeat purchases of organically or integrated fresh fruits and vegetables in Slovenia. The findings indicate that purchasing periodicity of organic fruits and vegetables is primarily influenced by income and product accessibility. Moreover, the regularity of purchasing organic fruit and vegetables is attributed to two quality attributes of a food product (tasting and visual attractiveness) and consumers’ environmental issues [26,27].

Gracia and Magistris [28] used a model on Lancaster’s theory paired with the expected utility discrete choice model to determine the determinants affecting consumers’ choice of organic food in the United States. According to the findings, females, the young, and trained people are much more likely to purchase organic foodstuffs. Additionally, dietary qualities such as genuineness, veganism, and locally produced are essential factors that shape customers’ willingness to buy healthy products.

Rezende [29] analyzed factors explaining consumers’ choice of organic food in the USA using a model based on Lancaster’s theory integrated with the random utility discrete choice model. Results indicate that females, younger and more educated people are more likely to buy organic foods. Food attributes such as vegetarianism and local production increase consumers’ probability of purchasing organic foods.

Yin et al. [30] surveyed Chinese consumers and developed a logit model to evaluate the fundamental factors affecting consumers’ organic food purchases. The findings show that income, level of faith in organic food, level of acceptance of organic food pricing, and consumers’ self-health concerns all substantially impact Chinese consumers’ desire to purchase organic food.

Consumers’ interest in the sustainability of food production and willingness to ban are factors influencing their preference for pesticide-free sustainable products. Blanco-Penedo et al. [31] investigated the elements influencing consumers’ buying of sustainable products without agrochemicals. The authors developed a clustering framework to identify the essential factors in selecting pesticide-free ecological food.

Regarding the second kind of research, for example, Suh and Eves [32] explored Korean consumers’ opinions and attitudes regarding organic food and identified factors that influence organic food selection. According to the study, consumers’ positive and negative thoughts, trust, and experiences all play a role in buying organic food.

Using focus groups and laddering interviews, Wezel et al. [2] investigated the significant factors related to organic fruit and vegetable choices in the United Kingdom. They concluded that health was the most crucial factor in buying organic food. However, they highlighted that socioeconomic forces, including supporting local farming, fair trade, and ecological benefits, play a significant role in organic buying habits.

To explore consumers’ preferred decisions on the value attribute of ecological food, Yin et al. [33] developed a Comprehensive Evaluation Index with numerous alternatives. According to the findings, consumers prefer food safety and hedonistic value qualities and a lesser priority for critically evaluating environmental value attributes. Consumers’ age, education level, and concern about environmental protection have a minor impact on their ecological protection intent.

Teng and Wang [34] investigated consumer views of organic-certified agricultural standards (CAS) and evaluated customer willingness to pay a premium for Fresh Milk when it has an organic CAS-certified label. Exploratory factor analysis examined primary factors of respondent perceptions and preferences. Fresh Milk Logo, price/promotion, organic, and product/brand were the four primary factorial variables retrieved from consumer consumption preferences for fresh milk. The factors determining WTP include “Fresh Milk Logo” and “organic”, and respondents are willing to spend an additional USD 21.95 per year for organic CAS milk. Finally, while there has been little empirical research in Ecuador on organic goods and consumers, it has yet to precisely examine socioeconomic aspects influencing consumers’ organic food choices.

### Determinants of Organic Demand and Consumer Constraints

Income and information on a product’s environmental qualities are two major factors influencing consumer purchasing decisions. Organic goods are generally more labor-intensive, produced on a smaller scale, and/or manufactured using more environmentally friendly processes, which might explain their higher price [35]. Although organic items are typically more expensive than their counterparts, budgetary constraints play a significant role in consumer decisions between organic and conventional products.

Furthermore, Xie [36] illustrates that “when customers are still unable to determine a product’s environmental performance, the price must be distorted upward to signal a clean product.” In a survey on the effectiveness of labeling environmentally certified forest food, Refs. [37,38] revealed that when two items have comparable environmental seals, customers “assume that the environmental attribute of the greater product is better.” Because of their low income, consumers are likely to organic items but prefer less expensive standard ones [39]. The growing competition for shallow price alternatives exacerbates this phenomenon.

Alternatively, the wealthiest buyers can get their preferred things quicker, which may or may not be eco-friendly. Consumers seem limited by a lack of awareness of the environmental implications from the cradle to the grave [40]. For instance, product life cycle ecological information is rarely included [41]. Consumers must search for, locate, and interpret such information as a response. This process could be lengthy, costly, and uncertain [42]. Even if a product contains environmental information, consumers cannot always interpret it. Due to original organic labeling, consumers can know that the branded product is better for the environment and nutrition over its whole life cycle. The organic label could also be beneficial in revealing consumers’ healthy preferences [43].

Consumers are concerned about nutrition and want to buy more environmentally friendly items due to the increased demand for organic products requiring organic labeling. Several academic fields highlight the central determinant of organic demand: ecological and nutritional consciousness, which is explained on the one hand by a certain degree of altruism and, on the other hand, [44], results in a willingness to pay more for an organic product.

## 3. Material and Methods

### 3.1. Methodology for Data Collection

#### 3.1.1. Study Area

**Figure 1:**
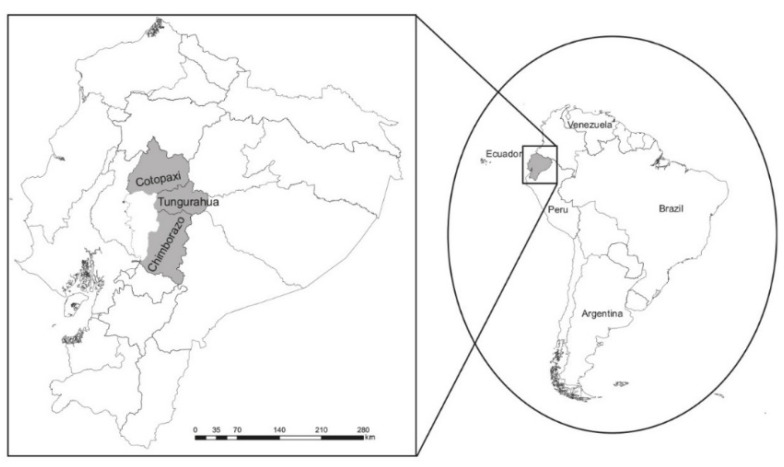
Location of the study area in Ecuador. Source: Authors’ representation based on [7].

More than 513,000 people live in the central region (see Figure 1). Arable land accounts for 13% of the total land area. Small farmers account for nearly all farms (0.1 to 5 ha). PACAT (Union of Agroecological Producers and Associative Marketing of Tungurahua) is the leading organization providing fresh organic food. PACAT has 350 members, with 75 percent of them being women. The primary distribution market is in Ambato City. A standard transaction process is direct selling or non-intermediary commercialization. As a result, by producing a range of fruits, vegetables, grains, and cereals, this sector (organic farming) contributes to local food security and sovereignty. The empirical study of this zone is a good example and illustrates the current state of the Ecuadorian organic food market.

#### 3.1.2. Survey Design and Sample Selection

A survey to gather the necessary information to apply was designed with questions in English, translated to Spanish (respondents’ native language), and checked by three experts to ensure that all terms were easy to understand. We pilot-tested the survey through exploratory interviews with five organic food consumers. Cronbach’s alpha coefficient validated the survey. The final survey consists of two major sections. The first section captures demographic characteristics, and the second collects information on purchasing and consumption aspects.

The study population consists of all consumers belonging to the following nine cities: Ambato, Baños, Cevallos, Mocha, Patate, Quero, Pelileo, Pllaro, and Tisaleo, registered by the PACAT records. In cooperation with a group of technicians from the Ministry of Agriculture, we randomly selected 422 consumers for the initial sample. The final sample included 382 consumers. The sample distribution is described in Table 1.

#### 3.1.3. Data Collection

In September 2021, we applied the survey to the consumers’ sample. We collected 382 questionnaires via face-to-face interviews. The interviewees were household heads. The survey asked residents about their perceptions of the organic products industry as well as their level of knowledge and awareness regarding organic food. We assessed citizens’ opinions and impressions of the organic products sector. The survey addressed the general public’s impression of the organic food sector. Based on these findings, we conducted reliability and validity analyses.

#### 3.1.4. Research Hypothesis and Variables Setting

Consumers’ motivations for buying organic food and their decisions can be explained by intricate functions influenced by various circumstances. Because of the disparities in consumers’ geography and culture, these motives and intentions are particularly atypical.

The following hypothesis is formed in light of the actual circumstances in Ecuador throughout the study’s interview phase: consumers purchase organic food for eight reasons. The respondents were asked to check the three most important reasons from a list of twelve during the survey. The study’s eight response options are: lacks chemicals from manufacturing or processing; tastes better; is healthier; is fresher; looks better; is better for the environment; supports farmworker health; is more natural. Meanwhile, the following are some of the reasons why consumers refuse to buy organic food: too expensive; inconvenient to buy; lack of knowledge about organic food; limited variety and brands; challenging to compare and choose; distrust of organic food; does not taste better than conventional food; does not believe traditional food is healthier.

The purchase frequency, site of purchase, consumption frequency, consumption, information level, age of consumers (age), education level (education), annual income (income), and gender are the nine characteristics that influence Ecuadorian consumers’ purchase behavior toward organic food. A radar diagram represents the percentage of respondents who checked a particular reason. Table 2 shows the socioeconomic and consumption characteristics of surveyed consumers.

### 3.2. Methodology for Analysis

#### Logit Model

This study focuses on customers’ purchase intentions or their willingness—or lack thereof—to purchase organic food. Consumer purchasing intent is influenced by nine elements, as previously stated. As a result, the following equation describes the relationship between buy intention and the nine factors: consumers’ purchase intention of organic food = f(gender, education, age, income, etc.) + random disturbing factor. In this paper, consumers’ move to purchase organic food is a 0–1-type dependent variable (when purchasing organic food, y = 1; otherwise, y = 0). Assuming that the probability y = 1 is P, the function y is as follows:

(1) $$f\left(y\right)=P^{y}{\left(1-P\right)}^{1-y},\;y=0,1$$

This work uses the logit model of binary choice, limits the number of dependent variables to [0,1], and computes the regression parameter using the maximum likelihood estimation method. The basic form of the logit model is as follows:

(2) $$P_{i\;}=F\left(\alpha \sum^{m}\beta_{j}X_{ij}+u\right)=1/\left\{1+\exp\left[-\left(\alpha +\sum^{m}\beta_{j}X_{ij}+u\right)\right]\right\}$$

where $P_{i}$ is the probability of i, which is the serial number of consumer, $\beta_{j}$ is the regression parameter of influencing factors, j is the serial number of influencing factors, m is the number of influencing factors, $X_{ij}$ is the independent variable representing influencing factor  j in sample i, α is the intercept and u is the error.

## 4. Sociodemographic Data of Respondents

Consumer behavior and socioeconomic variables have been connected in previous studies, e.g., [45]. Consumers’ age and education can influence their willingness to participate in organic food initiatives [46]. Consumers with more education prefer environmentally friendly items [47], whereas traditional consumers choose more traditional products [48,49]. We must first understand how demographic factors influence consumer choices to determine trade-offs. Income, purchasing location, and information are the attributes the customer considers. Appendix A presents the correlation between the analyzed variables. Table 3 shows descriptive statistics of the sociodemographic and organic food consumption characteristics of respondents.

## 5. Results and Discussion

### 5.1. Awareness Rate

According to the findings, Ecuadorian consumers are only 53.5 percent aware of organic food (53 percent in Ambato, 52 percent in Baos, and 55 percent in Pelileo). This share is much lower than the rate in wealthy countries, which has risen to as high as 80%. The rate of consumer awareness for organic food, in particular, varies significantly. We consulted customers if they had ever heard of organic food on a fundamental level. The rate of awareness described before measures this level. The second level entails consumers’ understanding of the relevant eco-labels and the leading certification authority, which is essentially the only way to determine the validity of organic food. Only 22% of consumers know one or more of these products. This share could be attributable to the Ecuadorian government’s lack of interest in the label. The ability of consumers to identify organic food from conventional, green, and non-harmful food in terms of quality and safety is the final level. A total of 31 percent of respondents (15.7 percent of the total participants) know the difference between quality and safety.

### 5.2. Willingness to Eat Organic

**Figure 2:**
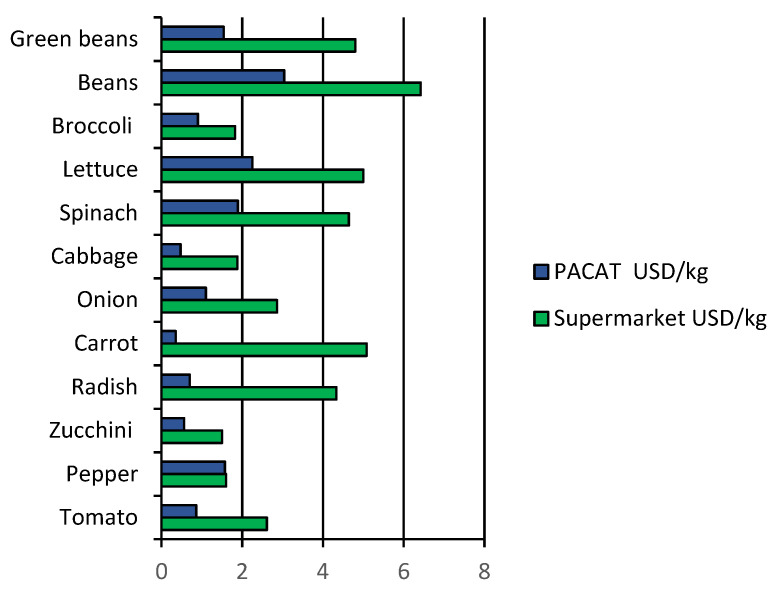
Comparison of the price per kilogram of organic products by seller type. Source: Workshop with sector stakeholders.

According to the findings, most customers are eager to eat more organic than conventional food (Table 4). The average willingness to consume (WTC) organic food is 75.3 percent higher than traditional food, matching research findings from European countries. However, the WTC level stays lower due to the market price of organic food, which is two to three times higher than conventional food. Figure 2 displays a price comparison between the supermarket and PACAT for organic products per kilogram (Ecuador’s most significant organic producers association). Based on the considerable gap between current pricing and consumers’ WTC, organic food will likely occupy a small share of regular consumers’ consumption. The WTC for various organic food categories varies according to the food categories studied. Fruits and vegetables have a relatively high WTC, but grains and milk products have a lower WTC. This aspect has to do with the fundamental qualities of food. Consumers are more concerned about food safety and are willing to pay more for foods consumed frequently, have high taste requirements, and have less chemical residue.

Concerning the price of organic food, 24% of consumers believe it is costly, 55% believe it is somewhat expensive, and only 16% believe it is the correct price. These shares indicate that price is relatively high and is inconsistent with Ecuadorian consumers’ current income levels. Thus, organic food prices have become a significant issue limiting the market in Ecuador.

### 5.3. Reasons for Choosing or Refusing Organic Food

**Figure 3:**
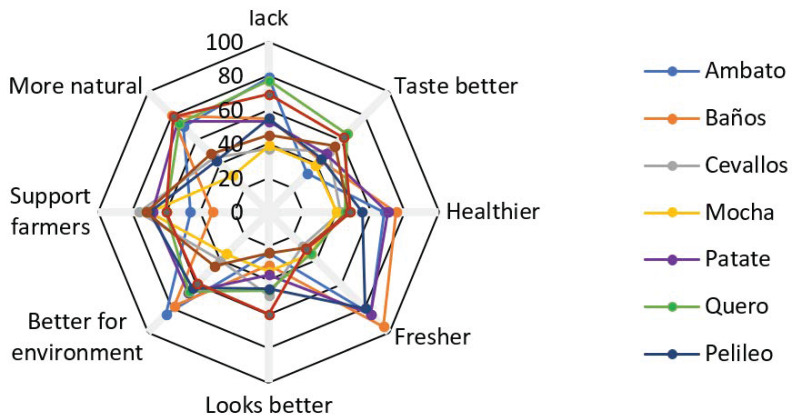
Motives why consumers choose to consume organic food % of respondents per city. Source: Authors’ own representation. Note: *p*-value of sample mean < 0.01.

**Figure 4:**
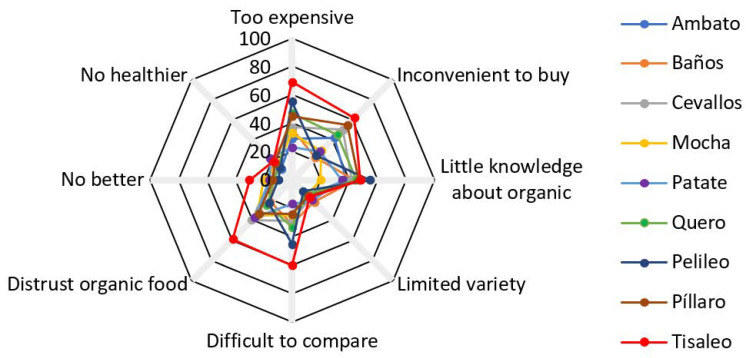
Motives why consumers refuse to consume organic food % of respondents per city. Source: Authors’ own representation. Note: *p*-value of sample mean < 0.01.

Respondents were asked to check each appropriate item from a list of six possible justifications during the survey. A total of 67.5 percent of respondents stated that the perceived lack of chemical content is the primary reason for purchasing organic food. The words “healthier”, “tastes better”, and ”better for the environment” (Figure 3). When it comes to not buying organic food, 70.5 percent of customers say it is “too pricey”. “Distrust of organic food” and “lack of information about organic food” are two other causes (Figure 4).

### 5.4. Logit Regression Analysis

We entered all variables into the regression equation to examine the regression coefficient, resulting in model 1. The variable with the lowest Wald value is then removed, and another regression is run until all variables are statistically significant. Model 2 outperforms Model 1 in terms of explaining the relevance of variables. The following discussion will concentrate on Model 2 (Table 5).

The age (OR = 1.01; 95% CI = [0.93, 1.09]) and education level (OR = 0.68; 95% CI [0.62, 0.73]) of consumers influences consumption intentions. Generally, the younger the consumer, the more likely they are to buy organic food. Young people are open to new experiences and have a sophisticated consumption mindset. Despite their refined consumption, the younger generation’s limited purchasing power and lack of care for their health offset their intense eagerness to acquire products. Furthermore, the study found that education impacts consumption intention, implying that well-educated customers or those knowledgeable about healthy eating are lured to organic foods. Results show that men have a more favorable attitude (directly related to their lifestyle) to the purchase and consumption of organic food than women, whereas women are inclined to pay a higher price for organic food than men. Meanwhile, their income level (OR = 1.002; 95% CI [0.88; 1.05]) positively influences customers’ propensity to eat organic food. Because organic food is a high-priced consumable, socioeconomic status has become a critical determinant affecting demand.

Organic food knowledge (OR = 0.662; 95% CI [0.58; 0.71]) influences consumer consumption intentions. Their knowledge of it determines consumers’ propensity to buy organic food. A greater understanding of organic food leads to a stronger desire to consume it. On the other hand, consumers’ concerns about the amount of available information regarding organic food and their shopping frequency substantially impact their consumption intention. No measures, according to respondents, ensure the consumer that the food is genuinely organic. We noticed the misapplication of the “organic product” seal on products that do not have it. Furthermore, consumption frequency (OR = 1.175; 95% CI [1.10; 1.27]) has a positive impact on organic food consumption intentions. Consumers vary in their frequency of organic food purchases (OR = 0.547; 95% CI [0.52; 0.57]), from a relatively small proportion who purchase it regularly to many more who have never purchased it. Respondents claimed that organic food (such as vegetables and fruits) has a two- to three-day shelf life and that they bought modest portions more frequently to take advantage of the food’s nutritional qualities.

### 5.5. Cross-Location Comparison

Table 6 shows the one-way ANOVA employed to determine differences between socioeconomic and information variables for the nine locations considered within the study. A multiple comparison test complements the findings by ranking statistically similar sectors.

Results for the socioeconomic and information variables show significant differences (*p* < 0.001) between the nine locations. Consumers from Ambato (the city with the bigger organic food market) have higher education and income levels and obtain more information about organic food and its benefits. This is in line with Girard and Rebaï [50], who assures that the number of short market circuits in this city has increased significantly in the last year. Likewise, there is a high intervention of public entities that promote this type of sales channel, which tries to connect the producer with the consumer without intermediaries.

Consumers from Baños, Patate, and Pelileo, the prominent touristic locations within the central region, have a higher income. According to Hidalgo-Crespo et al. [51], the per capita consumption of organic food in Baños has a growth rate of 17.5% per year, surpassing the consumption rate of Quito, the country’s capital. Patate and Pelileo are neighboring cities of Baños, and the consumption dynamics are similar in the three localities. Foreign tourists in these locations require a significant supply of organic food to meet the demand.

Consumers from Quero and Tisaleo have the lowest purchasing frequency of organic food. According to Bonisoli et al. [52], these localities lead the production of organic food such as vegetables and fruits. For example, in Quero, the area with the highest organic production of cape gooseberries, a fruit with high calcium and vitamins, is considered a superfood. Thistle, for its part, has led the organic output of tubers through agreements subject to research programs in which several regional universities have participated.

#### 5.5.1. Implications for Practice

These findings indicate that age and education influence buying organic foodstuffs. A greater understanding of organic food leads to a stronger desire to consume it. Organic food knowledge influences consumer consumption intentions. The socioeconomic level is also relevant to managing organic consumption decisions. Because organic food is a high-priced consumable, socioeconomic status has become a critical determinant affecting demand. Proper information management will foster organic and healthy food consumption and guarantee compliance with sustainability goals.

#### 5.5.2. Research Limitations and Future Work

A limitation of the study was that the findings were based on data collected from a single respondent (household head). We minimized the effects of the single respondent data collection; we collected the data from prominent representatives who would have the most knowledge about the household budget to buy food. Using multiple respondents or qualitative data would be an essential future extension to this research to evaluate the effect of socioeconomic variables on the willingness to consume organic food. Logit models cannot represent random variation in preferences, have restrictive substitution patterns, and cannot use panel data. Finally, the study expands knowledge about organic food consumption dynamics by exploring insights from the Ecuadorian context.

## 6. Conclusions

Understanding consumers’ intentions and willingness to consume organic food are essential to put public–private strategies into motion in Ecuador. This research presented a background of previous studies in which different frameworks are used to explain people’ willingness to consume. Based on a probit model, the study showed that age significantly negatively affected consumption intentions. The direct effect of consumption frequency was the largest. Further findings indicated positive effects, first from education, and second, from information level.

The findings can help policymakers understand the drivers of consumers’ willingness to consume organic food. Given the positive and significant impact of consumption frequency on consumers’ willingness, it is essential to improve trade areas to customers to influence their intention and engagement. The current promotion of organic food consumption is mainly based on the White Book of the Short Circuit Markets of the Republic of Ecuador. The draft takes the healthier diets principles as a baseline for national agri-food policy, provides a political context, and assigns specific responsibilities to government agencies and stakeholders. However, the impact of organic food consumption strategies and policies should be supported by funds that stimulate green and environmentally friendly supply. Tax exemptions could, for instance, incentivize the reduction of chemical agriculture by sharing experiences of organic agriculture among farmers. The government should do more to develop organic agri-food chains, for example, by coordinating initiatives such as reusing crop residues, animal manure, or crop rotation.

## Figures and Tables

**Table 1:** Sample distribution in the province.

Place	Proportion %	Sample
Ambato	65.4	249
Baños	4.0	15
Cevallos	1.6	6
Mocha	1.3	6
Patate	2.6	9
Quero	3.8	15
Pelileo	11.3	43
Pillaro	7.6	30
Tisaleo	2.4	9
Total	100.0	382

Source: Authors’ survey.

**Table 2:** Socioeconomic and organic food consumption characteristics of surveyed consumers.

Variables	Description	Measure
Gender	Sex or sexually orientation	male = 0, female = 1
Age	Age of household head	years
Education level	Last level of education	years
Income level	Range of family income	USD/month
Information	Knowledge about organic food properties	Perception (Likert scale 1 to 5)
Consumption	Consumption of organic food (0/1)	No = 0, yes = 1
Consumption frequency	Frequency of consumption per family	times/week
Place of purchase	Description of market, supermarket or fairing	informal = 0 formal = 1
Purchase frequency	Frequency of purchase per family (1–4)	times/week

Source: Authors’ survey.

**Table 3:** Mean, standard deviation, and *p*-value of sociodemographic and organic food consumption characteristics of respondents.

Variables	Unit	Mean	Proportion	S.D.	Max	*p*-Value Robust ANOVA
Age						
15–25	Years	17		2.3	25	0.058 *
25–45 years	Years	28		5.7	45	0.045 **
45–65 years	Years	51		3.5	65	0.028 **
more than 65 years	Years	68		9.4	74	0.039 **
Gender						
Male	Share		55.9		1	
Female	Share		44.1		1	
Education level						
Primary	Share		12.4		1	
Secondary	Share		48.1		1	
College	Share		39.5		1	
Income level						
Less than 400	USD/month	325		25.6	400	0.001 ***
400 and 1000	USD/month	830		88.5	1000	0.021 **
More than 1000	USD/month	1580		134.8	1700	0.027 **

Note: Differences in (p) represents the *p*-value significance of nine population with unequal sample and unequal variances: *** for 0.01, ** for 0.05, and * for 0.1. Source: Authors’ survey.

**Table 4:** Share (%) of consumers per city presenting WTC organic food categories.

City	Cereals	Fruit and Vegetables	Meat	Poultry and Egg	Milk Products
Ambato	45.7	63.4	57.5	59.6	39.6
Baños	30.2	52.6	49.3	40.1	44.1
Cevallos	27.8	50.1	30.4	26.7	17.5
Mocha	13.5	36.7	35.1	30.6	19.2
Patate	25.7	45.6	50.5	55.4	28.7
Quero	16.2	22.9	39.8	20.7	15.4
Pelileo	22.6	59.7	40.6	60.2	26.2
Píllaro	19.1	28.5	19.2	16.8	20.9
Tisaleo	15.9	20.2	25.9	10.7	21.3
Average	24.1	42.2	35.8	35.6	25.8

Source: Authors’ survey.

**Table 5:** Relationships between socioeconomic and consumption characteristics and likelihood of organic food consumption.

	Model 1				Model 2			
Variable	Coefficient	Wald	OR	95% CI	Coefficient	Wald	OR	95% CI
Age	−0.015 *	7.029	1.020	(0.97, 1.13)	−0.013 *	12.960	1.014	(0.93, 1.09)
Gender	−0.046	0.105	0.955					
Education	0.220 *	0.328	0.980	(0.88, 1.06)	0.225 **	6.055	0.685	(0.62, 0.73)
Income	0.010 *	9.016	1.010	(0.92, 1.08)	0.011 *	11.766	1.002	(0.88, 1.05)
Information level	0.267 **	1.346	1.306	(1.17, 1.42)	0.306 **	5.026	0.662	(0.58, 0.71)
Consumption frequency	0.722 **	2.971	1.161	(1.09, 1.25)	0.653 **	7.233	1.175	(1.10, 1.27)
Purchasing place	−0.031	0.092	0.872					
Purchasing frequency	−0.625 **	5.525	0.535	(0.51, 0.55)	0.603 **	5.230	0.547	(0.52, 0.57)
Constant	1.936 **	4.230	0.928	(0.89, 0.96)	1.720 **	10.024	0.584	(0.52, 0.63)
Prediction accuracy		67.4				66.2		
−2Log-likelihood		527.330				531.084		
Significance (*p*)		0.000				0.000		

** Correlation is significant at the 0.01 level (2-tailed). * Correlation is significant at the 0.05 level (2-tailed). Source: Authors’ own representation.

**Table 6:** ANOVA results and multiple comparison tests for socioeconomic and consumption characteristics of respondents.

		Tukey Test Multiple Comparison								
	F-value	Ambato	Baños	Cevallos	Mocha	Patate	Quero	Pelileo	Píllaro	Tisaleo
Age	757.46 ***	S1 = 45.6	S2 = 32.2	S3 = 25.1	S3 = 24.9	S2 = 35.1	S3 = 27.2	S2 = 34.2	S1 = 42.4	S3 = 28.2
Education	416.77 ***	S1 = 15.5	S1 = 14.3	S2 = 10.5	S2 = 11.2	S2 = 10.3	S2 = 9.5	S2 = 9.7	S2 = 10.8	S2 = 11.9
Income	591.83 ***	S2 = 520	S1 = 722	S2 = 493	S2 = 412	S1 = 671	S2 = 455	S1 = 730	S1 = 711	S2 = 403
Information level	729.55 ***	S1 = 4.5	S1 = 4.2	S3 = 2.5	S3 = 2.1	S1 = 4.1	S3 = 2.5	S3 = 2.3	S3 = 2.1	S2 = 3.1
Purchasing frequency	504.12 ***	S1 = 3.4	S2 = 2.2	S2 = 2.3	S2 = 2.5	S1 = 3.7	S3 = 1.5	S2 = 2.7	S1 = 3.1	S3 = 1.3

Note: *** denotes a coefficient significant at 0.001 level, Si is a statistically different sector. Source: Authors’ own representation.

## Data Availability

The data are available from the corresponding author.

## Appendix A. Correlation between Variables of Socioeconomic and Purchasing Preferences

	**Information**	**Consumption**	**Frequency**	**Place of Purchase**	**Gender**	**Age**	**Educational Level**	**Income Level**
Information	1	0.235 **	0.214 **	0.269 **	0.008	0.015	0.235 **	0.02
		0	0	0	0.876	0.769	0	0.703
Consumption	0.235 **	1	0.660 **	0.563 **	0.036	0.145 **	0.077	−0.086
	0		0	0	0.487	0.004	0.133	0.092
Frequency	0.214 **	0.660 **	1	0.495 **	0.013	0.128 *	0.049	−0.069
	0	0		0	0.795	0.013	0.339	0.181
Place of purchase	0.269 **	0.563 **	0.495 **	1	0.013	0.032	0.095	−0.059
	0	0	0		0.798	0.532	0.064	0.247
Gender	0.008	0.036	0.013	0.013	1	0.051	0.017	−0.066
	0.876	0.487	0.795	0.798		0.319	0.733	0.195
Age range	0.015	0.145 **	0.128 *	0.032	0.051	1	0.098	−0.004
	0.769	0.004	0.013	0.532	0.319		0.056	0.931
Educational level	0.235 **	0.077	0.049	0.095	0.017	0.098	1	0.264 **
	0	0.133	0.339	0.064	0.733	0.056		0
Income level	0.02	−0.086	−0.069	−0.059	−0.066	−0.004	0.264 **	1
	0.703	0.092	0.181	0.247	0.195	0.931	0	
	382	382	382	382	382	382	382	382
Source: elaborated by the authors. * Correlation is significant at the 0.05 level (2-tailed), ** Correlation is significant at the 0.01 level (2-tailed).								
